# Bindings for Action: Bridging the Gap Between Theories of Procedural Working Memory and Action Control Research

**DOI:** 10.5334/joc.488

**Published:** 2026-02-12

**Authors:** Gidon T. Frischkorn, Isabel Courage, Hannah Dames, David Dignath, Christina U. Pfeuffer, Moritz Schiltenwolf, Andrea Kiesel, Klaus Oberauer

**Affiliations:** 1 Institute of Psychology, University of Zurich, Zurich, Switzerland; 2 Institute of Psychology, University of Freiburg, Freiburg, Germany; 3 Institute of Psychology, University of Tübingen, Tübingen, Germany; 4 Institute of Psychology, Catholic University, Eichstätt-Ingolstadt, Germany

**Keywords:** action control, BRAC, procedural working memory, binding

## Abstract

Two research traditions, action control and procedural working memory research, have addressed the question of how humans control actions, largely independently from each other. While both research traditions consider binding as an important mechanism for action control, they conceptualize this mechanism differently. Here, we argue that a comparison and synthesis of both research traditions might lead to a better understanding of the conceptualization of bindings. We first provide a brief overview of recent frameworks developed in the respective research traditions: the BRAC framework (Frings et al., 2020) and the procedural working memory (WM) model (Oberauer, 2013). We then analyze the similarities and differences between the BRAC and WM perspectives. As a first step toward fostering an integrative model of action control, we present an extension of the procedural WM model that can account for the main empirical findings investigated within the BRAC framework and that might serve as a blueprint for future integration.

Many of our everyday activities require us to process information quickly and select appropriate actions to achieve our goals. Consider driving: when a traffic light turns from green to red, we must rapidly shift our attention and press the brake pedal to stop the car. This example illustrates a central research question: How does the human cognitive system identify relevant stimuli, selects suitable actions, and establishes the correct links between them?

The selection and execution of goal-directed actions have been studied, largely in parallel, within two separate research traditions: action control and working memory (WM). These traditions have employed different theories, taxonomies, and paradigms. This separation is regrettable, as both traditions share the core assumption that actions are controlled by representations that bind together relevant aspects of the situation with corresponding responses. Here, we call for increased communication between these disciplines, suggesting that research from both fields would benefit from an integrative perspective.

The present article takes a step in this direction by examining two influential frameworks: the Binding and Retrieval in Action Control framework (BRAC; Frings et al., 2020) and the declarative and procedural WM model (Oberauer, 2009). We outline common principles and key differences through a systematic comparison of both accounts with the goal of bridging these traditions to advance a unified theory of human action control. As an initial step, we apply the procedural WM model to an experimental setting typically investigated within the BRAC framework.

Theories of action control aim to explain how stimuli and actions become linked through learning or instruction. In the BRAC framework (Frings et al., 2020), aspects of the situation—that is, representations of stimulus features—and actions—that is, representations of motor behavior, often referred to as responses—are integrated into so-called event files. The concept of event files originates from the Theory of Event Coding (Hommel, 2004; Hommel et al., 2001), which posits that stimulus, response, and contextual features of an action episode are bound together into a common representation. When a feature from a previous episode reoccurs, the corresponding event file is retrieved, reinstating other features that were previously bound. In this way, the retrieval of past event files influences action selection and control in the current episode, providing a mechanism for how previous experiences shape ongoing behavior.

Bindings between elementary representations are also central to many theories of WM. Research in this tradition has largely focused on declarative memory, that is, representations of entities and their relations in the world. Examples include the serial order of words in a list, spatial locations, and the different features of objects. In the context of action control, we focus on theories of WM that include procedural memory, representing what to do in a given situation (see Monsell, 2003). For instance, Oberauer’s (2009) model conceptualizes declarative and procedural WM as two subsystems within a unified WM architecture, sharing analogous structures and operating principles. Whereas declarative WM maintains representations of the current situation, procedural WM binds these representations to corresponding actions, enabling goal-directed behavior.

Both research traditions have inspired numerous empirical studies examining when and how information is bound and retrieved. The BRAC framework emphasizes the binding of responses into event files, whereas research on WM has resulted in a computational model that captures the formation, maintenance, and retrieval of bindings (see Oberauer et al., 2013; see also Haazebroek et al., 2017; Schmidt et al., 2016, 2020, for computational models of action control). Drawing parallels between the theoretical foundations of the BRAC framework and procedural WM accounts offers an opportunity to advance our understanding of goal-directed actions. By clarifying how each tradition conceptualizes the encoding, maintenance, and retrieval of procedural bindings, we aim to identify common principles as well as critical differences. We are convinced that synthesizing insights from these two complementary domains will not only deepen theoretical accounts of action control research but also shed light on the representational format of bindings in WM (see Kaup et al., 2024).

To this end, we first review both perspectives before analyzing their theoretical overlap, focusing on similarities and differences in representations and processes. Based on this analysis, we conjecture that integrating insights from both traditions will foster a more unified understanding of action control. As a first step toward integration, we exemplify how an extension of Oberauer’s (2009) procedural WM model can account for a typical result pattern observed within the BRAC framework.

## Introduction to the Theoretical Foundations

### Binding and Retrieval in Action Control (BRAC)

The *Binding and Retrieval in Action Control* (BRAC; Frings et al., 2024; Frings et al., 2020) framework builds on ideomotor approaches to action control, which propose a common representation of perception-related and motor-related codes—a principle referred to as “common coding” (Hommel et al., 2001). According to ideomotor theory, movements are not initiated by dedicated motor programs but rather by representations of the anticipated effects or outcomes they produce. These effects include proximal feedback, such as proprioceptive, sensory information about the position and movement of one’s own body as well as distal feedback, such as perceptual, emotional, or social consequences (e.g., Eder, 2023; Kunde et al., 2018; Shin et al., 2010). When planning an action, the activation of the intended effect representations automatically activate the corresponding motor behavior. This account of action control requires bidirectional bindings between stimulus features and motor representations. Ideomotor learning establishes bindings between activated representations of motor behavior and resulting proprioceptive and distal effects. Action planning starts with the activation of the intended effects, which automatically retrieves the corresponding motor representation (e.g., Dignath et al., 2014; James, 1890; Kunde, 2001; for a historical overview of the ideomotor theory, see Stock & Stock, 2004).

In contrast to other perspectives on perception and action (e.g., Goodale & Milner, 1992; see also Creem-Regehr & Kunz, 2010, for an overview of different perspectives on perception and action), BRAC assumes the same representational format for perception and action. Accordingly, both percepts (e.g., stimuli and effects) and motor behavior are represented by features such as shape, location, or movement and are integrated into an event file/episode (e.g., Frings et al., 2024). Interestingly, models on action control only partly incorporate these assumptions. For example, HiTec (Haazebroek et al., 2017) assumes a common format for perception and action, yet does not assume event files. In contrast, the PEP model by Scmidt and colleagues implements an episode layer, yet does not assume the same representation format for stimuli and responses (Schmidt & Liefooghe, 2016; Schmidt et al., 2020).

**Figure 1:**
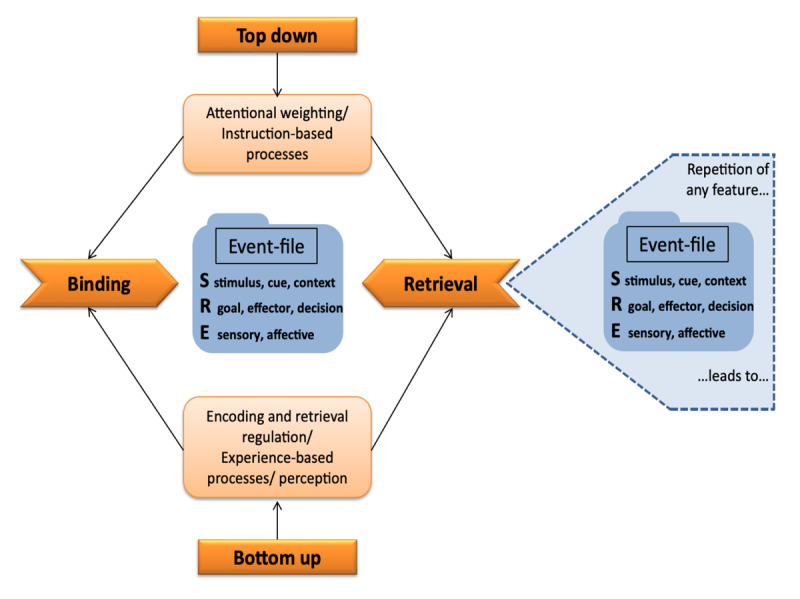
Illustration of the Binding and Retrieval in Action Control (BRAC) framework. *Note*. Sketch of the BRAC framework. Adapted from Frings et al., (2020).

BRAC proposes two distinct processes that are central for action control (see Figure 1): The binding of feature representations of an episode, referred to as event files, and the subsequent retrieval of event files when at least one feature of the event file re-occurs. The binding process constitutes an integration of feature representations of an episode into an event file (Hommel et al., 2001). Thereby, only features that are currently activated can be bound and the amount of activation of the features determines the probability that the respective features become bound (Hommel et al., 2001). As a result of binding, an event file comprises neuronally distributed, multimodal feature representations that refer to the experienced distal properties of the event (in contrast to proximal representations like the neuronal firing pattern on the retina representing a visual stimulus; Hommel, 2019; Hommel et al., 2001).

The retrieval process describes the automatic activation of all feature representations of an event file when at least one of the bound features is re-encountered (Hommel, 2004). Retrieval facilitates performance if the features of the present episode fully overlap with the retrieved event file, but impairs performance if only some of the previous features repeat, resulting in impaired performance in partial repetition conditions (Hommel, 1998). Unbinding costs and confusion due to the retrieval of mismatching features have been discussed as reasons for these so-called partial repetition costs (Frings et al., 2024; Mocke et al., 2023). Such partial repetition costs dissolve relatively fast suggesting that corresponding bindings have a short lifespan either due to passive decay or due to an active unbinding process (Frings et al., 2022; Hommel & Frings, 2020).

Binding and retrieval processes are assumed to depend on top-down mechanisms, such as attentional weighting or instructions, and bottom-up processes, such as regulation or the experience-based impact on processes or perception (see Figure 1). The BRAC framework successfully accounts for phenomena in various action-control paradigms, including stimulus categorization tasks (Hommel, 1998; Rothermund et al., 2005), action planning (Kunde et al., 2002; Stoet & Hommel, 1999), task switching (Koch et al., 2018; Schiltenwolf et al., 2024; Schmidt & Liefooghe, 2016), and sequential effects in Stroop-like tasks (Dignath et al., 2019; Hommel et al., 2004; Spapé & Hommel, 2008). The implications of BRAC are quite general and address different paradigms in experimental psychology. Since the repetition of a single feature bound in an event file leads to its retrieval, BRAC predicts that behavior in the current trial is influenced by the previous trial whenever features repeat.

In the BRAC framework, event files are conceptually similar to instances as specified in the instance theory of automatization (Logan, 1988, 1990). Both event files and instances are presumed to be incidentally formed due to the co-occurrence of event features and to be automatically retrieved when features reoccur (Frings et al., 2020, 2024; Logan, 1988, 1990). Whereas event files are usually examined with regards to their short-term impact on behavior, research inspired by instance theory also demonstrated long-term effects of instance formation on performance (Logan, 1988). For example, the longevity (spanning several trials or even minutes) of binary stimulus-response bindings formed by single-trial item-specific priming (e.g., Moutsopoulou et al., 2015; Pfeuffer et al., 2017, 2020) contrasts with the short-term nature of bindings according to BRAC (e.g. Frings, 2011; but see Moeller & Frings, 2021). These discrepancies highlight the importance of a better understanding of the role of memory in the formation and maintenance of bindings.

### Procedural Working Memory

When Miller, Galanter, and Pribram (1960) introduced the concept of WM, they defined it as a representational medium for action plans. Later research on WM retained the conceptual emphasis on both the short-term maintenance and processing of information. However, the focus of most empirical research and theorizing has shifted toward the mechanisms of maintenance of declarative representations (e.g., Baddeley, 2012; Cowan, 2005; Ma et al., 2014).

An attempt to conceptualize the mechanisms of the “working” aspect of WM was provided by the framework proposed by Oberauer (2009). A core assumption of this framework is that there are two analogous subsystems within WM: declarative and procedural WM. The procedural subsystem serves as a representational medium for representations of intended actions and as a mechanism for action control. In this context, actions are defined as intentionally bringing about a change either in one’s physical environment (i.e., overt actions) or in one’s mental representations (i.e., cognitive actions). For our comparison, we will focus on procedural WM and its overlap with and differences from the theoretical assumptions of the BRAC framework.

**Figure 2:**
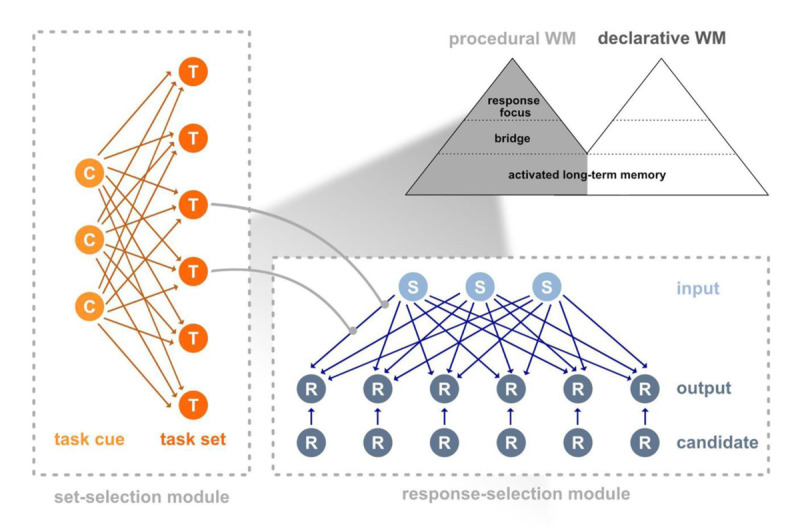
Illustration of the procedural WM model and its main components, the set-selection and response selection module. *Note*. Procedural WM is a WM subsystem next to declarative WM. The model developed by Oberauer et al. (2013) consists of two modules, the set-selection module and the response-set module. In the set-selection module the current task cue (C) activates the task set (T) currently relevant. The response-selection module holds the task-relevant bindings between stimuli (S) and responses (R). The activated stimuli are represented in the input layer which forward activations to the responses in the output layer. The model contains an additional third layer, the candidate layer, which is a replica of the output layer.

According to Oberauer (2009), representations in procedural WM are condition-action rules that guide overt or cognitive actions. They can be expressed as “if-then” statements, such that if a condition is fulfilled when input is provided (e.g., if an object is red), then a specific action is performed (e.g., then press a certain button). This subsystem consists of three embedded components: the activated part of long-term memory (LTM), the bridge, and the response focus (Oberauer, 2009, 2010; see Figure 2).

Although less broad than the BRAC framework, the procedural WM model has also been applied to action control, particularly in contexts that require the implementation and/or switching of stimulus-response mappings (often referred to as task sets; e.g., Formica et al., 2020; Liefooghe et al., 2012; Meiran & Cohen-Kdoshay, 2012; Oberauer et al., 2013; Souza et al., 2012). To formalize its core theoretical assumptions, Oberauer et al. (2013) developed a connectionist network model (see Figure 2). The architecture of the procedural part of the model includes two modules: the set-selection module and the response-selection module. The set-selection module identifies the task set that is relevant for the upcoming action, whereas the response-selection module selects the required response from the activated task set (Oberauer, 2020; Oberauer et al., 2013).

Conceptually, the set-selection module belongs to the activated LTM component, whereas the response-selection module represents the bridge (Oberauer, 2009; Oberauer et al., 2013) with a limited set of procedural bindings—that is, bindings between conditions and to-be-executed actions—that form the currently relevant task set from which the to-be-given response is selected. Within this framework, selecting a response from the currently active task set is assumed to occur in a manner akin to a “prepared reflex”—that is, a fast and automatic response selection once the task set has been established (Exner, 1879; Hommel, 2000; Kiesel, 2009; Logan, 1978; Oberauer, 2020; Oberauer et al., 2013).

The response-selection module is implemented as a two-layer network and contains an input layer, which represents the stimulus conditions (e.g., target stimuli), and an output layer, which represents the corresponding responses (e.g., the action of pressing a specific button). Stimuli and responses are modeled as localist representations, meaning that each is encoded by the activation of a dedicated unit with the respective layer. Bindings in this model are instantiated through connections between the input and the output layer, with a weight matrix representing all learned bindings (Oberauer, 2020; Oberauer et al., 2013). Please note that in contrast to this architecture – which clearly separates stimulus and response representations, the BRAC framework assumes the same coding format for stimulus and response features and thus bindings in the same representation format (Frings, et al, 2020). However, only the HiTec (Haazebroek et al., 2017), but not the PEP model (Schmidt & Liefooghe, 2016; Schmidt et al., 2020) incorporates this assumption.

When a task set is encoded in procedural WM via the response-selection module, attention is directed to stimuli and stimulus features that are relevant for that task. This prioritizes the activation of representations of relevant stimulus information in the input layer (Oberauer, 2010). Activation in the input layer is forwarded to the output layer through the procedural bindings to activate one or several response representations, the strongest of which is selected for execution (Oberauer, 2009).

During and after response selection, when stimulus and response representations are simultaneously activated in their respective layers, they are bound together by updating the connection weights in the weight matrix through Hebbian learning according to the delta rule. When there are any discrepancies between the expected target output and the actual target output based on the current input, the delta rule rapidly updates the weight matrix. The Hebbian learning process applies to every representation that is simultaneously active. Hence, target stimuli as well as distractors are bound to responses within these procedural bindings to the degree that they are attended to. While forming new bindings in the bridge occurs with a rapid learning rate, parallel slower learning of associations between stimuli and responses takes place in the activated part of LTM (Oberauer et al., 2013).

To switch between bindings from different task sets, the set-selection module holds multiple learned task sets. This module reflects another aspect of the activated part of LTM in which task sets that are currently not relevant are maintained and can be retrieved later (Mayr & Kliegl, 2000; Oberauer, 2009; Oberauer et al., 2013). Similar to the response-selection module, this module has a two-layer architecture and consists of a cue layer and a set layer. The task cues in the cue layer are each bound through connections to the set layer where each learned task set can be represented as a pattern of activations (Oberauer et al., 2013). Activating a task cue leads to activation of the associated task set in the set layer. From there, it is read out into the response-selection module to update its weight matrix. However, updating is never complete, so that a residue from previous bindings remains and causes interference with the current bindings (Oberauer et al., 2013).

In summary, this theoretical framework of procedural WM assumes that procedural representations are implemented as bindings between stimuli and responses, represented in different components of WM—more permanently in the activated LTM and more momentarily in the bridge. Hence, the relevant task set can be retrieved from LTM into WM, and the required responses can be selected in WM to guide actions. According to this theory, facilitation and interference effects between previous and current action selection come about not through the retrieval of previous action events but rather through the continuing impact of activated representations, fast-changing bindings, and slow-changing associations in the network. Thus, the model also accounts for trial-to-trial carryover effects that are central to action control research, such as switching costs, automatic effects of instructions, and sequential dependencies between actions. As we show later these effects arise naturally from the time required to update task sets and from the incomplete overwriting of previously active stimulus–response bindings.

## Comparative analysis of BRAC and WM theories

Building on the preceding introductions to the BRAC framework and the procedural WM model, we now turn to a systematic comparison of these accounts. While, for example, their similar perspectives on the formation of stimulus-response bindings, demonstrate conceptual commonalities between these theoretical frameworks, they also diverge in key aspects. In the following section, we evaluate their theoretical connections and examine the extent to which their proposed representations and processes assumed in both theories converge. Likewise, we aim to identify aspects that are uniquely addressed by one theoretical framework but could possibly enrich the other if integrated. Finally, we establish contradictions between the two frameworks to point out components that need to be reconciled or more rigorously tested in future research.

We have organized this comparative analysis of the BRAC and WM theories into four parts: 1) the general system structure they propose, 2) the assumed structure and properties of bindings, 3) the proposed processes and how they act on bindings, and 4) their practical application with respect to experimental designs and results. A summary of this comparison can be found in Table 1 at the end of this section.

**Table 1:** Summary of the comparative analysis of BRAC and WM perspectives on the representation of stimulus-response bindings.


		BRAC FRAMEWORK	WM

**General System Structure**	Separation of Memory Systems	Single memory system with short-lived bindings; recent clarifications suggest transition from WM to LTM based on binding strength, repetition, and spacing.	Separation of WM (limited capacity, fast learning rate) and LTM (large capacity, slow learning rate, stores information permanently). LTM representations built in parallel to WM.

	Capacity Limits	Implicit capacity limits assumed, based on decay and interference within WM.	WM has limited capacity; LTM has no apparent capacity limit. Interference in WM as a limiting factor.

	Representation of Stimuli, Responses	Event files with neuronally distributed, multimodal feature representations; no clear distinction between perception and action.	Stimuli and responses represented as activation patterns in distinct layers.

	Representation of Task, and Control Sets	Task and control states included but not specifically detailed.	Task sets are represented as bindings in the response-selection module, with learned sets in the set-selection module.

**Structure and Properties of Bindings**	Formation of Bindings	Created based on co-occurrence, influenced by attention and saliency; bindings of all features in independent event files.	Rapid Hebbian learning binds active representations, represented in a weight matrix across events; strength influenced by activation levels. Allows for independent pairwise bindings between stimuli and responses.

	Episode Boundaries for Bindings	Bindings exist for an episode, with boundaries under investigation (decay and unbinding debated).	Bindings remain until actively removed; no causal role for episode boundaries.

	Code Occupation	Feature representations can only be in one event file at a time; unbinding needed for new bindings.	No constraint on binding features to multiple stimuli or responses.

	Sequential Effects on Bindings	Short-lived binding effects; full repetition benefits vs. partial repetition costs.	Previous actions affect subsequent actions due to temporary strengthening of stimulus-response bindings, incomplete updating of bindings, and residual activation.

**Proposed Processes**	Selective Attention	Selective attention influences which features are bound; activated features have higher binding probability.	Features activated through attention are bound via Hebbian learning; selective attention modulates activation strength.

	Learning	Bindings as building blocks for learning; transition to LTM with binding strength.	Separate rapid binding in WM and slow learning in LTM; parallel updates in response-selection and set-selection modules.

	Retrieval	Feature repetition serves as retrieval cues; reactivates the entire event file.	Cue-based retrieval; stimuli act as retrieval cues for responses; task cues act as retrieval cues for task sets.

	Updating	Not explicitly specified; dissolution through decay or unbinding necessary for new bindings.	Explicit updating through delta rule; iterative process until match criterion is reached.

**Practical Application in Experiments**	Dependent Variables	Primarily reaction times, with some accuracy measurements; focus on sequential effects in prime-probe designs.	Equal weighting of reaction times and accuracy; general model for action control in simple tasks.

	Application in Paradigms	Broad application across various paradigms (e.g., stimulus categorization, action planning, task switching, Stroop-like tasks).	Applied to task-set switching, memory-set switching, and object switching within memory sets.


### General system structure

#### Separation of memory systems

In BRAC, binding of features into episodic event files is presumed to rely on a single memory system that has so far mostly not been further specified. However, the short lifespan of bindings observed in experiments inspired by the BRAC framework suggests that this memory system does not correspond to LTM. More recently there have been attempts to clarify the memory systems that event files are stored in according to BRAC (Frings et al., 2023). According to this idea, event files are initially stored in WM and are subject to decay and interference over time unless reinforced through repetition or retrieval processes. The transition of these event files from WM to LTM is theorized to depend on factors such as repetition, spacing, and the strength of the initial binding.

The WM framework separates bindings in WM (represented by the response-selection module) from bindings in LTM (represented by the set-selection module, and the long-term associations between stimuli and responses). WM is responsible for the manipulation and maintenance of bindings in an easily accessible state, whereas LTM stores bindings more inflexibly over extended periods, allowing for retrieval when needed. Apart from the specific causes that underlie forgetting of bindings or event files, the assumptions of BRAC align with a general separation of memory systems in the WM framework. One critical difference is that in the WM framework, LTM representations are built up in parallel to WM representations, whereas in the newly proposed separation of memory systems from the BRAC perspectives, LTM representations are built once the strength of bindings in WM exceeds a certain threshold.

In addition, it is important to distinguish slowly learned stimulus–response associations in the procedural working memory framework from event files as conceptualized in the BRAC framework. In procedural WM, long-term stimulus–response associations cannot be used directly to select a response for execution and therefore cannot bypass the bridge (i.e., the response-selection module). Instead, long-term associations can only bias or prime response activation; response selection itself is always mediated by the currently active task set instantiated in procedural working memory.

Moreover, the representational separation between stimuli and responses is maintained in long-term memory as well as in working memory. Information flow in the procedural WM theory is strictly directional from stimulus to response, both in WM and in LTM. Unlike BRAC, the procedural WM model does not assume common coding between perception and action: responses do not act as retrieval cues for the stimuli they are associated with, and the retrieval of long-term associations does not reinstate holistic action episodes. Thus, even when multiple stimulus–response associations are jointly retrieved into the bridge, they remain componential associations whose influence on behavior is constrained by the currently active task set, rather than forming episodic representations akin to BRAC event files.

#### Capacity Limits

With respect to capacity limits of the different memory systems, the WM framework assumes WM to be limited in capacity, whereas LTM has no apparent capacity limitation. Although the computational model by Oberauer et al. (2013) implements interference between bindings in WM as one limiting factor of WM capacity, the specifics of how interference limits the capacity to maintain bindings are not fully implemented. The BRAC framework, having not committed to a clear separation of memory systems yet, does not specify if and how memory capacity is limited with respect to the storage and maintenance of bindings or event files. If anything, capacity limits have been included implicitly through the assumptions that event files should be stored in WM and are subject to decay or interference.

#### Representation of stimuli and responses

According to BRAC, an event file consists of neuronally distributed, multimodal feature representations that are associated with each other. The feature representations refer to the experienced distal properties of the event (in contrast to proximal representations like the neuronal firing pattern on the retina representing a visual stimulus; Hommel, 2019; Hommel et al., 2001). Whether bindings are binary or configural is still under investigation and might depend upon, for instance, the saliency of features or configurations (Frings et al., 2024; Moeller et al., 2016). As BRAC derives from ideomotor theories of action control that emphasize a common representational form of action and perception (common coding; Hommel et al., 2001), actions are presumed to be represented by their perceivable proximal or distal effects (e.g., Eder, 2023; Kunde et al., 2018; Shin et al., 2010). Thus, BRAC does not posit a representational distinction between perception and action, because both are assumed to be represented by features such as shape, location, or movement (e.g., Frings et al., 2024).

In the WM model, stimuli and responses are represented as activation patterns in the stimulus and the response layer, respectively. The model does not explicitly specify whether these representations represent proximal or distal entities. However, the applications of the model to experiments illustrate that these representations are meant to represent distal entities such as the identity of a letter or the location of a response in space, rather than proximal entities such as line orientations in a stimulus and motor commands for a response. In the computational model, these representations are currently implemented as localist codes. However, this is a choice of convenience rather than a theoretical commitment. So far the model has not been applied to experiments in which there was a need to distinguish multiple features in stimuli or responses, but such an extension would be straightforward. In fact, other models of declarative WM (Oberauer et al, 2012; Kowialiewski & Oberauer, 2025) have used distributed representation for different feature dimensions that are similar to the representations proposed by BRAC. Unlike BRAC, the WM model distinguishes between stimuli and responses as they are represented in separate layers. Additionally, in the WM model there is currently no representation of action effects separately from representations of the actions (responses) themselves. Action representations are activated through stimulus representations, not through anticipations of action effects.

To conclude, BRAC emphasizes the commonality between representations of perception (“stimuli”, situation) and intended actions (“responses”) (“common coding”), while the procedural WM model stresses the functional separation between declarative knowledge and procedural rules.

#### Representation of task and control sets

With respect to task and control states, BRAC generally assumes their existence to account for sequential effects in task switching and congruency sequence effects (Benini et al., 2022; Dignath et al. 2019; Schiltenwolf et al., 2024). Thereby BRAC states that such task and control states encompass mental representations and stimulus dimensions that are relevant for the current task, including response options and stimulus-response mappings. However, BRAC does not specify how they are stored and under which circumstances they are activated to guide behavior.

The WM framework, in contrast, explicitly states that the currently operative task set is represented as a set of bindings between the two layers of the response-selection module. In addition, other learned task sets are represented as patterns of activation in the set-selection module. These can replace the currently activated task set in response to a task cue through updating the response-selection module. This updating is, however, often incomplete leading to interference from previously activated task sets.

### The structure and properties of bindings

#### Formation of Bindings

According to BRAC, bindings evolve whenever features co-occur. Single co-occurrence (Hommel, 1998; Frings et al., 2007; Rothermund et al., 2005) and even verbally instructing co-occurrence appears to be sufficient to establish bindings (e.g., Liefooghe et al., 2012; Pfeuffer et al., 2017) Thereby, the formation of bindings depends on, for instance, attention (Moeller & Frings, 2014; Memelink & Hommel, 2013) or saliency (Schmalbrock et al., 2021; Qiu et al., 2022). That is, only features that are currently attended will be bound into the event file, whereas unattended features or context information that is not sufficiently represented will not be bound into the event file.

In the WM model, bindings are created through rapid Hebbian learning. Hence, to the extent that distractors or context information are attended to and thereby activated in the stimulus or the response layer, they are bound together. In the computational model, the speed of learning is set by the learning rate and the strength of the binding is a function of the activation of different features. Consequently, features that are less activated, for instance, due to attentional filtering of distractors, will build up weaker bindings than features that get full attention and are thus more strongly activated.

Taken together, the formation of bindings according to BRAC and WM perspectives is conceptualized rather similar, with two differences. First, in BRAC bindings form event files in which the features of the event are bound, whereas in procedural WM there are only pairwise bindings between stimulus features and responses, and not between stimulus features (these could be represented separately in declarative WM). Second, in BRAC, the unit of memory is an episode, and individual episodes are stored as separate traces (as in instance theories of memory), whereas in procedural WM, memory traces of individual episodes are superimposed (i.e., added up) in the changes of the weight matrices.

#### Episode Boundaries for Bindings

In BRAC, bindings in an event file are presumed to be formed for feature co-occurring in an episode. Yet, what constitutes the event boundaries of an episode is still under investigation. For instance, contextual changes are discussed as such boundaries (Qiu, et al., 2023). Event files created in different episodes are generally considered independent, unless there is overlap in features which requires the dissolution of previous event files. In the WM model, bindings are represented in the weight matrix and result from currently active task sets and from Hebbian learning during responding. The end of an episode (e.g., a trial) does not by itself impact on bindings. More generally, there is no causal role for the beginning or end of an episode in the WM model.

#### Code Occupation

According to BRAC, feature representations can only be included in a single binding or event file at any time (Stoet & Hommel, 1999; but see also Mocke et al., 2023). That is, as soon as a feature, say the color red, is associated with a response, then the same feature cannot be associated with another response without dissolving the event file of the previous episode that the feature was included in. Correspondingly, partial repetition costs have also been explained as costs of unbinding to make feature representations available for the formation of new bindings. In the procedural WM model, there is no such constraint that a stimulus (or stimulus feature) can only be bound to one response (feature), or vice versa. Thus, the same stimulus can be bound to different responses in separate task sets. This does not lead to ambiguity because response selection is guided by the bindings of the currently implemented task set in the response-selection module.

#### Sequential effects on bindings

According to BRAC, previously formed bindings affect performance on a subsequent trial if some or all features of previous event files repeat (full repetition benefits vs. partial repetition costs). Thereby, binding effects are typically short-lived, and it has yet to be determined whether passive decay or active unbinding or both lead to the dissolution of event files and associated binding effects (Frings, 2011; Hommel & Frings, 2020; Frings et al., 2022). Thus, the exact lifetime of bindings and conditions influencing it are currently not yet well understood.

In the procedural WM model, previous bindings affect what happens in the next trial because previous bindings are not removed at the end of a trial or an episode. When the next trial requires a different set of bindings, the current stimulus-response bindings are updated through Hebbian learning, but this updating is not complete. Therefore, residual bindings from the previous trials keep affecting processes in the current trial. In addition, residual activation of stimuli, and response suppression, carry over from one trial to the next.

### Proposed Processes

#### Selective Attention

Selective attention plays an important role in both BRAC and WM theories, as it influences which features are bound and which are not. In BRAC, it is assumed that the activation of the features precedes binding (Hommel, 2019). Furthermore, the activation level determines the probability that a certain feature becomes bound (Hommel et al., 2001). The activation level in turn can be influenced by attention and/or saliency (Memelink & Hommel, 2013; Schmalbrock et al., 2021).

In the WM model, features are activated in the stimulus and the response layer to the degree they are attended to. Simultaneously active features in both layers are automatically bound together. Selective attention is not yet part of the computational WM model. However, it makes sense to follow the emerging consensus in the WM literature that attention to stimuli is a necessary – though probably not sufficient – condition for encoding that stimulus into WM. By analogy, the same is probably true for response options. Therefore, attention determines how strongly features are activated in WM. The strength of activation will in turn determine how strongly features of stimuli and responses are bound together.

#### Learning

In principle, both BRAC and WM theories assume that bindings represent the building blocks for learning and more permanent storage in LTM. In BRAC, recently there have been attempts to outline theoretical mechanisms underlying learning in the BRAC framework (Frings et al., 2023; Giesen et al., 2020). Specifically, it is assumed that event files are initially stored in WM. As bindings in an event file get strengthened, for example through repetition, they are learned more permanently once they exceed a certain threshold of binding strength. They are then transformed into long-term memory representations that are stored more permanently and can be retrieved when features of this LTM event-file repeat.

The procedural WM model implements more permanent learning into long-term memory in two ways: (1) In the response-selection module, simultaneously active stimuli and responses are associated with each other through an additional slow Hebbian learning process. This process operates on a second weight matrix separated from the (rapidly learned) binding weight matrix. And (2) the set of stimulus-response mappings represented in the rapidly learned binding weight matrix can be transferred as an integrated representation of a task set in the set-selection module (and associated to a task cue for later retrieval). Thus, unlike the BRAC framework in which LTM learning is a function of binding strength in WM, the WM model clearly separates rapid binding in WM from slow LTM learning of stimulus-response links. These two processes proceed in parallel, updating separate but parallel weight matrices connecting the two layers of the response-selection module.

#### Retrieval

In BRAC, repeating features (referring to both stimuli or responses) serve as retrieval cues for other features bound to them. Given the structure of event files, all features included in the event file should get reactivated during the retrieval. However, the details of the retrieval process are not further specified.

The WM model also relies on cue-based retrieval. However, unlike in BRAC, responses are not considered cues that can lead to the retrieval of other stimulus features. Therefore, in the response-selection module, only stimuli act as a retrieval cues for the response; retrieval means that the stimulus representation activated in the stimulus layer feeds activation to the response layer through the two weight matrices (one for WM bindings, the other for LTM associations). Similarly, in the set-selection module, task cues act as retrieval cues for integrated representations of task sets that then update the response-selection module to implement the task set for execution during the next response process.

#### Updating

BRAC does not include an updating process and instead suggests that if features of an existing event file partially overlap with features of the current episode, the old event file needs to be unbound, and a new one is created. In contrast, the procedural WM model explicitly specifies how task sets – that is, the stimulus-response bindings in the response-selection module – are updated. First, the new task set is retrieved as a pattern of activation in the set-selection module. Each unit in this activation pattern corresponds to one connection in the connection-weight matrix of bindings in the response-selection module. In this way, the activation pattern representing the new task set teaches the updating of the stimulus-response bindings in the response-selection module. This updating process is governed by Hebbian learning with the delta rule, allowing for simultaneous formation and removal of bindings. Thus, unlike BRAC, the WM model does not assume that bindings have to be removed prior to building up new bindings.

## Integrating insights from BRAC and procedural WM

While the BRAC framework and the procedural WM model both address action control and share many similarities, they also differ in some respects. We conjecture that an integrative perspective of both accounts will be fruitful for future research and for a better understanding of bindings for action. As a first step in that direction, we applied the computational version of the procedural WM model (Oberauer et al., 2013) to a prototypical experiment demonstrating partial repetition costs. Following this approach, different model versions could be built and applied to the empirical phenomena that are currently explained by the BRAC framework and the WM theory.

**Figure 3:**
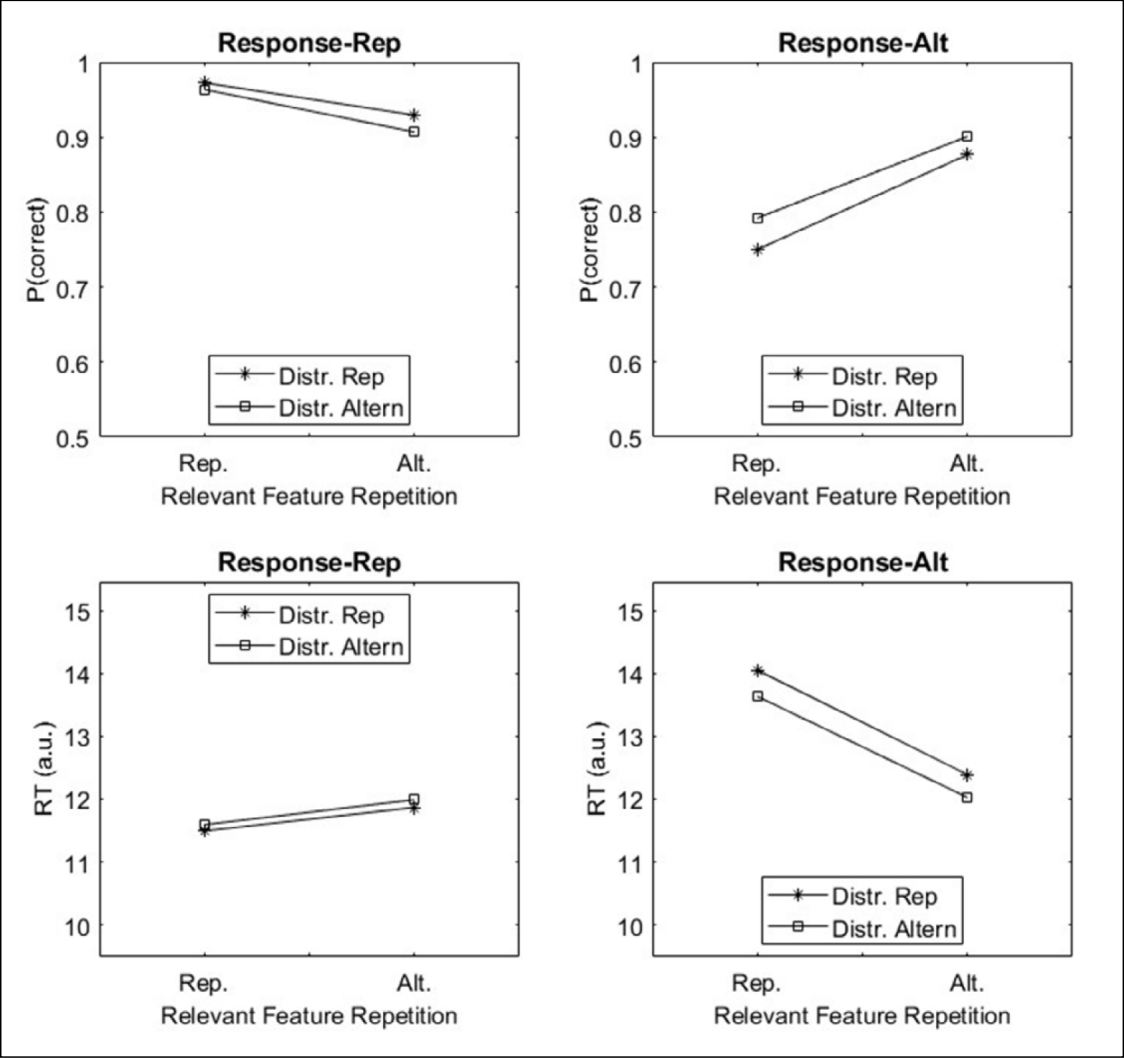
Predictions form the procedural WM model for partial and full repetition effects. *Note*. Data simulated with the WM model applied to an experiment demonstrating partial-repetition costs for relevant and irrelevant stimulus features as well as responses. Reaction times (bottom row) are simulated in an artificial unit (a.u.) and absolute values should not be interpreted. Rep = repetition; Alt = alternation.

In detail, we simulated an experiment with pairs of trials. In each trial, a stimulus with a relevant and an irrelevant feature was presented and a binary decision had to be made based on the relevant feature. Between trials, we varied repetition of the relevant feature, repetition of the irrelevant feature (distractor), and repetition of the response, mimicking standard experimental settings in BRAC (e.g., Frings, et al., 2007; Frings & Möller, 2010; Giesen, et al., 2012; Giesen & Rothermund, 2014; Hommel, 1998; Rothermund, et al., 2005). We applied the model as described in Oberauer et al. (2013) with a few modifications, the most important of which is the inclusion of the distractor feature in the stimulus representation, and a parameter for attentional filtering (set to 0.5) that dampens the activation of irrelevant relative to relevant features. Figure 3 shows simulated proportion correct and response time averages for the second trial. The simulated data show the common pattern of repetition effects: Performance is poorer with partial repetitions of one or two of the three components (relevant feature, distractor feature, or response) than with full repetition or full alternation.

As can be seen from the simulation results (see Figure 3), the computational model of pWM already captures basic effects in the domain of action control. Specifically, it predicts often observed costs – that is lower proportion correct (see top left row) and slower response times (see left bottom row) – of feature alternation from trial *n*-1 to *n* when responses repeat from trial *n*-1 to *n*, whereas it predicts benefits – that is higher proportion correct (see top right row) and faster response times (see bottom right row) of feature alternation from trial *n*-1 to trial *n* when responses alternate between trial *n*-1 and trial *n*. Based on these simulations, the next step could be to investigate which findings that support the BRAC framework cannot be reproduced by the WM model. On that basis, the pWM could be adapted by drawing on assumptions in BRAC to enable it to accommodate these findings as well. This has the potential to adjudicate conflicting assumptions between BRAC and the procedural WM theory and refine our understanding of how memory processes contribute to action control.

Different from BRAC, in the procedural WM model responses do not function as retrieval cues for other information. This might limit the model’s potential to explain the learning and execution of motor sequences. A plausible extension would be to represent novel motor sequences initially as ordered sets of elementary actions, each of which is bound to its ordinal position in the sequence, analogous to serial-order representations in declarative working memory. Through repetition these sequences could be chunked into unified action representations. Explicitly exploring how such sequence-level representations can be integrated into procedural WM remains an important topic for future work.

## Conclusion

Action control has been investigated by two separate lines of research. In this paper, we exemplarily analyzed similarities and differences of the BRAC framework (Frings, et al., 2020) and the procedural WM model (Oberauer, 2013). Both accounts agree that preparing and executing an action entails modification of bindings – either the formation of a new binding structure (event file) or the strengthening of existing bindings. Further, retrieval of information bound in previous action episodes in similar situations contributes to the representations controlling the current action. As a first step to integrate both accounts, we explored to what extent the computational model of procedural WM accounts for typical sequential effects of partial and full feature repetition in action control experiments typical in the BRAC framework. Given the promising results, this approach could serve as a blueprint for extending the procedural WM model to foster an integration of BRAC and procedural WM accounts of action control.
